# Wastewater-based Epidemiology and SARS-CoV-2: Variant Trends in the Apulia Region (Southern Italy) and Effect of Some Environmental Parameters

**DOI:** 10.1007/s12560-023-09565-0

**Published:** 2023-09-21

**Authors:** Francesco Triggiano, Osvalda De Giglio, Francesca Apollonio, Silvia Brigida, Fabrizio Fasano, Pamela Mancini, Giusy Bonanno Ferraro, Carolina Veneri, Giuseppina La Rosa, Elisabetta Suffredini, Luca Lucentini, Nicola Ungaro, Giuseppe Di Vittorio, Onofrio Mongelli, Nelhudoff Albano, Maria Teresa Montagna

**Affiliations:** 1 Interdisciplinary Department of Medicine, University of Bari Aldo Moro, Piazza G. Cesare 11, Bari, 70124 Italy; 2 Department of Biological and Environmental Sciences and Technologies, University of Salento, Campus Ecotekne, Monteroni di Lecce, Lecce, 73047 Italy; 3 Department of Environment and Health, Istituto Superiore di Sanità, Rome, 00161 Italy; 4 Department of Food Safety, Nutrition and Veterinary Public Health, Istituto Superiore di Sanità, Rome, 00161 Italy; 5 Agency for the Environmental Prevention and Protection (ARPA Puglia), Corso Trieste 27, Bari, 70126 Italy; 6 Department of Health Promotion and Animal Welfare, Apulia Region, Bari, Italy

**Keywords:** SARS-CoV-2, Wastewater, Wastewater-based epidemiology, COVID-19, Wastewater surveillance, Sewage

## Abstract

During the COVID-19 pandemic, wastewater monitoring has been used to monitor the levels of SARS-CoV-2 RNA entering the sewerage system. In Italy, the Istituto Superiore di Sanità coordinated the SARI project (Sorveglianza Ambientale Reflue in Italia) to detect SARS-CoV-2 and its variants. In this study, the concentration of SARS-CoV-2 and its variants in raw wastewater against COVID-19 cases was evaluated together with the effect of temperature and precipitation on virus spread. We validated a predictive model, proposed by De Giglio et al., 2021, to establish the number of COVID-19 cases/100,000 inhabitants. A receiver operating characteristic curve model was applied to predict the number of COVID-19 cases and Poisson regression was applied to study the effect of temperature and rainfall on viral load. In Apulia, from October 2021 to December 2022, we analyzed 1041 samples, of which 985 (94.6%) tested positive for SARS-CoV-2. Median atmospheric temperature was inversely proportional to viral load in wastewater; no correlation was found with precipitation. The predictive model confirmed that at least 11 cases/100,000 inhabitants would occur in the 15 days following the detection of the virus in wastewater. Environmental surveillance of SARS-CoV-2 can be used to map the virus and its variants.

**Supplementary Information:**

The online version contains supplementary material available at 10.1007/s12560-023-09565-0.

## Introduction

According to the World Health Organization (WHO), severe acute respiratory syndrome owing to SARS-CoV-2 resulted in 767,726,861 confirmed cases of COVID-19, including 6,948,764 deaths as of July 5th, 2023 (WHO, 2022). The pandemic has deeply impacted communities and health care systems worldwide, highlighting the need for continued efforts to fight the spread of the virus.

When individuals are infected with SARS-CoV-2, whether they are symptomatic or asymptomatic, the virus and its genetic components can be shed through various bodily excretions such as feces, urine, and saliva, as well as through respiratory products. These viral particles can reach sewage treatment plants via household wastewater generated during daily activities such as washing, bathing, showering, and using the toilet (Zheng et al., 2020).

During the COVID-19 pandemic, environmental surveillance through wastewater-based epidemiology (WBE) has been used to monitor the viral load and genetic diversity of SARS-CoV-2 in wastewater (Chassalevris et al., 2022; Yu et al., 2022). This type of surveillance complements clinical surveillance and facilitates the timely collection of data on community-level infections, assisting public health authorities in making decisions to control the spread of the virus (Hart & Halden, 2020; Pecson et al., 2021; Connie, 2023). In this regard, several studies (Sharkey et al., 2021; Yu et al., 2022; Zhan et al., 2022) have shown that the concentrations of SARS-CoV-2 RNA in wastewater are correlated with the number of COVID-19 cases in the catchment areas served by wastewater treatment plants (WWTPs).

Several variants of SARS-CoV-2 have emerged during the COVID-19 pandemic, characterized by substitutions and deletions in the spike protein (Chassalevris et al., 2022). With the spread of variants of interest (VOIs) and variants of concern (VOCs), some aspects of SARS-CoV-2 infections have changed, including transmissibility, disease severity, diagnostic methods, and treatment efficacy (Davies et al., 2021a, b; Wibmer et al., 2021; Jahn et al., 2022). To draw greater attention to the formation and transmission of SARS-CoV-2 variants, the European Commission advised EU Member States to set up monitoring of SARS-CoV-2 and its variants in wastewater by 1 October 2021 (European Commission, 2021). In Italy, the Istituto Superiore di Sanità (ISS) established the SARI (Sorveglianza Ambientale Reflue in Italia) network (from October 2021 to March 2023), enrolling 18 Italian regions and two autonomous provinces, to detect variations of SARS-CoV-2 and analyze its genetic diversity.

To date in Italy, several authors have already reported the results of SARI project (La Rosa et al., 2022, 2023; Cutrupi et al., 2022; Verani et al., 2022; Maida et al., 2023), but to our knowledge no studies have concerned the influence of environmental parameters on the virus load and the application of predictive models on the number of COVID-19 cases through the WBE. In a preliminary voluntary study (Apulia, southern Italy), De Giglio et al., 2021 propose a predictive model (De Giglio et al., 2021), which allowed us to hypothesize the occurrence of at least 11 cases COVID-19/100,000 inhabitants within 15 days after positive SARS-CoV-2 wastewater sampling.

The aims of this study were (i) to estimate SARS-CoV-2 concentrations in untreated wastewater with respect to the COVID-19 cases in the Apulia Region (Southern Italy), (ii) to investigate the trend in SARS-CoV-2 variants in wastewater, (iii) to evaluate the impact of environmental parameters such as temperature and rainfall on the load of SARS-CoV-2 in wastewater, and (iv) to verify the predictive model proposed in the previous study by De Giglio et al. (2021) with updated data sets concerning SARI project.

## Materials and Methods

### Study Design

Apulia region (southern Italy) extends for 19,541 km^2^ with about 4 million inhabitants. It is characterized by a typical Mediterranean climate, with mild, dry winters and hot summers. Rainfall is highly irregular throughout the year. Italian National Agency for the Environmental Protection reports that Apulia has some of the highest average temperatures across all regions of Italy; moreover, it is among the regions with more scarce rains (ISPRA, 2022).

At present the region is served by 187 WWTPs distributed among the six provinces: BA = 27; BT = 12; BR = 18; FG = 69; LE = 39; TA = 22, managed by the Apulian Aqueduct (AQP), which treat 74% of the pollutants produced by wastewater (De Giglio et al., 2021; Apulia Water Agency, 2023). Moreover, it is among the Italian regions with the highest number of WWTPs analyzed for SARI project (16 WWTPs), including Sicilia (17 WWTPs), Liguria (16 WWTPs), Lombardia (15 WWTPs) and Emilia Romagna (14 WWTPs).

### Selection of WWTPs and Sampling

During the period October 2021 to December 2022, we monitored 16 WWTPs, of which 12 were from municipalities with > 50,000 inhabitants and four from cities (Bari and Taranto) with > 150,000 inhabitants. Municipalities with more than 150,000 inhabitants were monitored twice a week, and those with 50,000–150,000 inhabitants were monitored weekly.

**Fig. 1:**
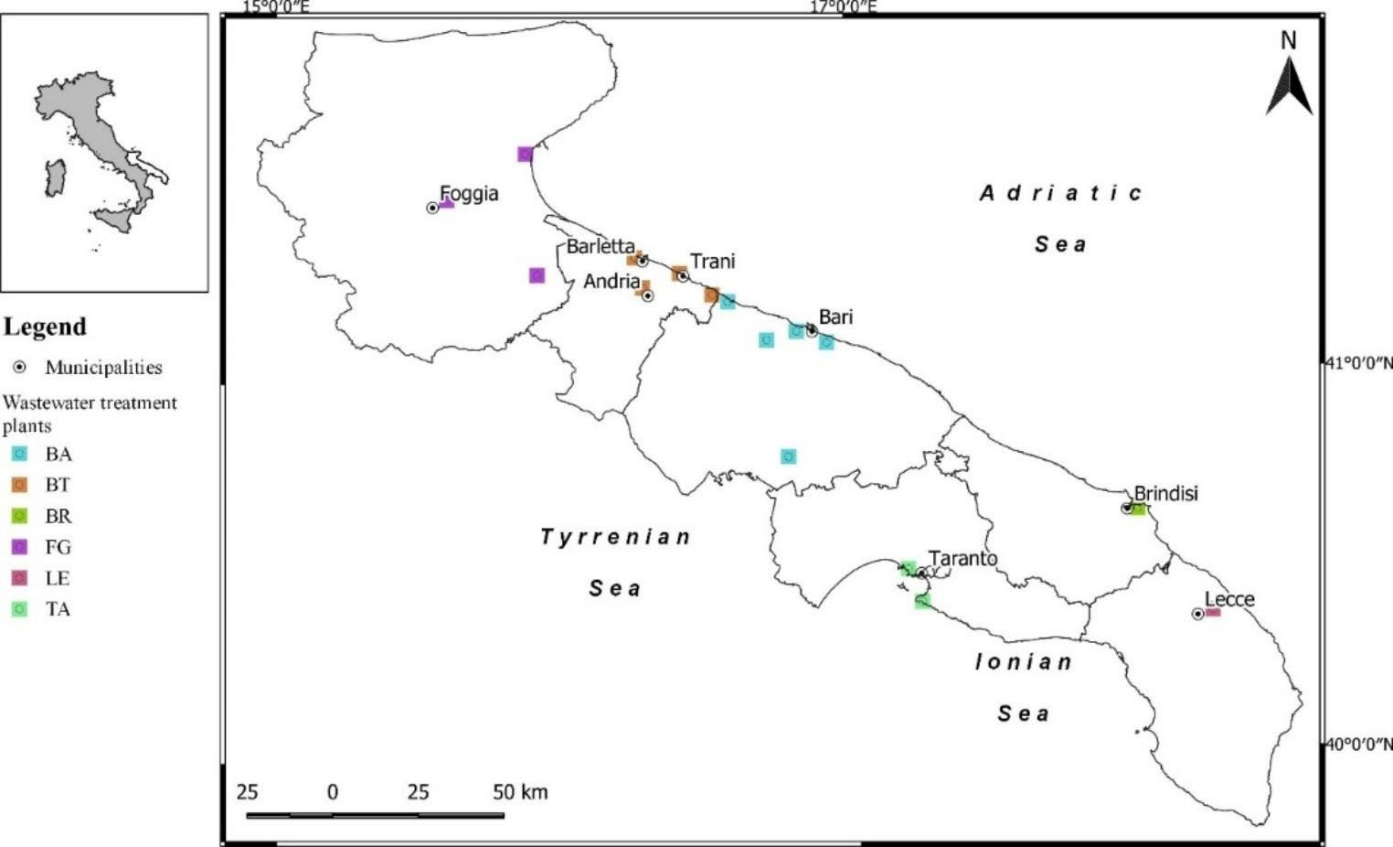
Location of enrolled wastewater treatment plants in the Apulia Region (Southern Italy) BA, Bari; BT, Barletta-Andria-Trani; BR, Brindisi; FG, Foggia; LE, Lecce; TA, Taranto

We selected the following WWTPs (identified with letters), which were equally distributed throughout the region (Fig. 1): BA with five WWTPs (A to E), BT with four (A to D), BR with one WWTP (A), FG with three (A to C), LE with one (A), and TA with two (A and B). Overall, 1041 samples of untreated wastewater were sampled by the Regional Agency for Environmental Prevention, in collaboration with the Apulian Aqueduct (AQP).

We collected 24-hour composite samples from the influent of WWTPs (1 L) prior to treatment using an automated sampler. Samples were stored at 4 °C and delivered in isothermal containers to the Environmental and Food Hygiene Laboratory-University of Bari Aldo Moro, the reference laboratory of the SARI Project in the Apulia Region. The laboratory analyzed the samples to detect and quantify SARS-CoV-2 (see Sect. Virus concentration and RNA extraction and Real Time reverse-trancription quantitative polymerase chain reaction (RT-qPCR)). The results were transmitted to the ISS via the SARI ARC-GIS platform within 48 h of sampling. Additionally, an aliquot of 50 mL of each sample was frozen and stored as an “archive sample” for any future confirmation tests or for further determination.

### Temperature and Rainfall Data

During the study period (October 2021 to December 2022), data on temperature and rainfall were obtained from the website of the Regional Civil Protection of Apulia (Apulia Regional Civil Protection, 2023). Specifically, the median atmospheric air temperature and the temperature range recorded 24 h before the day of sampling (during collection of 24-hour wastewater composite samples) were collected. Rainfall data were noted for 7 days prior to the day of sampling.

### Virus Concentration and RNA Extraction

To ensure comparable results, in compliance with the technical specifications outlined in EU Recommendation 2021/472, all participating centers in the nationwide SARI network implemented the same reference analytical protocol (ISS. SARI Rev.3 protocol). First, wastewater samples were subjected to thermal inactivation of SARS-CoV-2 at 56 °C for 30 min. After cooling, 45 mL of each sample was concentrated using polyethylene glycol (PEG), as described previously (Wu et al., 2021). To remove larger debris, the samples were centrifuged at 4500 × g for 30 min; subsequently, 40 mL of each sample was mixed with 8% (w/v) PEG 8000 and sodium chloride (0.3 M) (Sigma-Aldrich, St. Louis, MO, USA) and centrifuged at 12,000 × g for 2 h. A process control virus (murine norovirus, MNV-1 kindly provided by ISS) was added to each sample before PEG precipitation to monitor viral recovery from the samples.

RNA extraction was performed using the NucliSENS miniMAG semi-automated extraction system with magnetic silica (bioMerieux, Marcy l’Etoile, France), with slight modification of the manufacturer’s protocol (the lysis phase was extended, from 10 to 20 min) (De Giglio et al., 2021). Before molecular analysis, the eluted RNA was purified to remove residual PCR inhibitors using the OneStep PCR Inhibitor Removal Kit (Zymo Research, Irvine, CA, USA) and then stored at − 80 °C.

### Real-time Reverse-transcription Quantitative Polymerase Chain Reaction (RT-qPCR)

Each 25 μL reaction mix contained 5 μL of RNA, 12.5 μL of 2X reaction buffer supplied with AgPath-ID™ One-Step RT-PCR Reagents (Applied Biosystems, Foster City, CA, USA), 1 μL of 25X RT-PCR enzyme mix, 1 μL forward primer (12.5 μM), 1 μL reverse primer (22.5 μM), 1 mL probe (6.25 μM), 1.83 μL nuclease-free water (non-DEPC, treated with diethyl pyrocarbonate), and 1.67 μL of detection enhancer for real-time PCR (Applied Biosystems). The following primer and probe sequences were used: CoV-2-F: ACA TGG CTT TGA GTT GAC ATC T (code 2297); CoV-2-R: AGC AGT GGA AAA GCAT GTG G (code 2298); CoV-2-P: FAM-CAT AGA CAA CAG GTG CGC TC-MGBEQ (code 2299) (La Rosa et al., 2021). The real-time RT-PCR experiments were carried out in triplicate using the CFX96 Touch Deep Well Real-Time PCR System (Bio-Rad, Hercules, CA, USA) for detection and Quant Studio 12 K Flex (Applied Biosystems) for quantification. Thermal cycling conditions were reverse transcription at 50 °C for 30 min, reverse transcriptase inactivation at 95 °C for 10 min, and 45 amplification cycles at 95 °C for 15 s and 60 °C for 45 s (30 s when using Quant Studio 12 K Flex with the fast thermal profile). In the presence of RT-qPCR cycle threshold (Ct) values below 40, the samples were interpreted as positive for SARS-CoV-2 RNA. Ct are used as an indicator of SARS-CoV-2 RNA copy number.

In quantitative analysis, verification of inhibition and percentage recovery were performed for each sample, as reported by La Rosa et al. (2021). Reactions were performed in duplicate. For standard curve construction, the targeted region was synthetized and purified by BioFab Research (Italy) and quantified by fluorometric measure (Qubit, Thermo Scientific). Tenfold dilutions were used for standard curve construction (range 5 × 100–5 × 10^4^ copies/μL). An external amplification control consisting of in vitro synthesized RNA containing the target region was used to verify PCR inhibition. Amplifications were considered acceptable if inhibition was ≤ 50% and if standard curves displayed a slope between − 3.1 and − 3.6 and an R^2^ ≥ 0.98 (La Rosa et al., 2021).

### Investigation of SARS-CoV-2 Variants in Wastewater

As part of the environmental surveillance of SARS-CoV-2 in Italy, the ISS has been conducting national flash surveys since October 2021, i.e., periodic (monthly) sampling campaigns to investigate the virus variants. During the first week of each month, each laboratory in the SARI network submits the nucleic acids extracted from weekly samples to the ISS for evaluation of the genetic variability of SARS-CoV-2 via sequencing of a 1600-bp fragment of the spike region, using both Sanger sequencing and next-generation sequencing (La Rosa et al., 2021; La Rosa et al., 2022).

Information related to SARS-CoV-2 loads and variants in samples in which sequencing was possible is reported in monthly bulletins published by the Istituto Superiore di Sanità - Rome, Italy (ISS. Acque reflue, 2023).

### Statistical Analyses

#### Descriptive Statistical Analysis of SARS-CoV-2 Concentration and Trend in Variants in Wastewater

We performed descriptive statistical analysis considering the distribution and the results of samples from WWTPs, analyzed by province. The results relating to surveys carried out on wastewater with respect to COVID-19 cases that occurred in the study area were evaluated for the period October 2021 to December 2022.

#### Multivariate Analysis of SARS-CoV-2 Load in Wastewater Versus Environmental Parameters (Temperature and Rainfall)

Initially, the number of cases was correlated with the number of swabs test/day in the Apulia Region. This approach reduces the underestimation of COVID-19 cases associated with the variability of swab testing over time and across geographic areas (i.e., owing to testing hesitancy). Therefore, considering that the maximum daily number of COVID-19 swab tests carried out in the Apulia Region during the study period was 13,359, we used the following formula, where A represents the estimate of the number of COVID-19 cases in the served area on x day (to eliminate the change owing to the number of swab tests carried out on x day):

$$\begin{array}{l}{\rm{A = }}\,{\rm{No}}{\rm{.}}\,{\rm{of}}\,{\rm{COVID - 19}}\,{\rm{on}}\,{\rm{day}}\,{\rm{x,}}\,{\rm{in}}\,{\rm{the}}\,{\rm{area}}\,{\rm{served}}\,\\{\rm{by}}\,{\rm{WWTP}}\,{\rm{X}}\,{\rm{*}}\,\left( {{\rm{13,359/No}}{\rm{.}}\,{\rm{swabs}}\,{\rm{carried}}\,{\rm{out}}\,{\rm{on}}\,{\rm{day}}\,{\rm{x}}} \right)\end{array}$$

The estimated number of COVID-19 cases (A) in the population served by each WWTP, is defined by applying the following formula:

$${\rm{B = }}\,\left( {{\rm{A/population}}\,{\rm{served}}\,{\rm{by}}\,{\rm{WWTP}}\,{\rm{X}}} \right)\,{\rm{ \times }}\,{\rm{100,000}}$$

B indicated the number of COVID-19 cases/100.000 inhabitants in the population served by each WWTP. This formula allowed to “normalize” the data of number of cases in areas with different populations making them comparable.

Multivariate analysis of the viral load in positive samples is applied by Poisson regression model. In particular, the viral load is compared with (1) the number in COVID-19 cases during the 15 days prior and following sampling; (2) the population served by each WWTP; (3) the average daily flow (m^3^/d) in each WWTP; (4) the geographic location of each WWTP; (5) the median atmospheric air temperature in the 24 h prior to sampling; (6) the temperature range in the 24 h prior to sampling; (7) the amount of rainfall in the 7 days before sampling.

One parameter requiring special attention in the analysis was the geographic area of each WWTP. Because this parameter was originally descriptive in nature, it had be converted into a comparable alphanumeric format for inclusion in the Poisson regression model. To accomplish this, the ordinal coding technique (Potdar et al., 2022) was used. This involved assigning a progressive order to the WWTPs, based on their location from north to south across the Apulia Region.

The following formula of normalization was used to standardize the various units of measurement of independent parameters (Fasano et al., 2021):

$${\rm{x}}\,{\rm{normalized = }}\left( {{\rm{x}} - {\rm{x min}}} \right){\rm{/}}\left( {{\rm{x}}\,{\rm{max}} - {\rm{x min}}} \right)$$

x normalized = normalized data.

x = set of observed values for each record of analysis.

x min = minimum values of x.

x max = maximum values of x.

The final model is made by those variables with a p-value of < 0.05, as determined in the preliminary model of all variables.

The predictions in the final model of Poisson regression for each studied parameter were used to compute an overall risk score (relative risk) to quantify the effects of the above parameters on viral load using the technique used in other studies (Conza et al., 2013; De Giglio et al., 2019).

The correlation between the viral load in the positive samples and rainfall data was investigated using the Wilcoxon rank-sum test with continuity correction.

#### Validation of a Previous Predictive Model

We validated a predictive model that had been proposed previously (De Giglio et al., 2021), which allowed us to hypothesize the occurrence of at least 11 cases/100,000 inhabitants within 15 days after positive SARS-CoV-2 wastewater sampling. We calculated sensitivity and specificity using the data collected for this paper. Similar results would validate and strengthen the results of the developed model.

Statistical analysis was performed using R software version 4.0.5 (The R Project for Statistical Computing, Vienna, Austria), and a p-value < 0.05 was considered statistically significant.

## Results

### SARS-CoV-2 Concentrations in Untreated Wastewater

SARS-CoV-2 RNA was detected by quantitative real-time PCR in 94.6% (985/1,041) of untreated wastewater samples. The median viral load was 6350 genome copies/liter (g.c./L) (range 28–975,000) (Table 1). Among the different provinces studied, LE had the highest median levels of SARS-CoV-2 RNA (12,200 g.c/L), followed by BT (8800 g.c./L), BR (6600 g.c./L), FG (6340 g.c/L), TA (5600 g.c./L), and BA (5250 g.c./L).

**Fig. 2:**
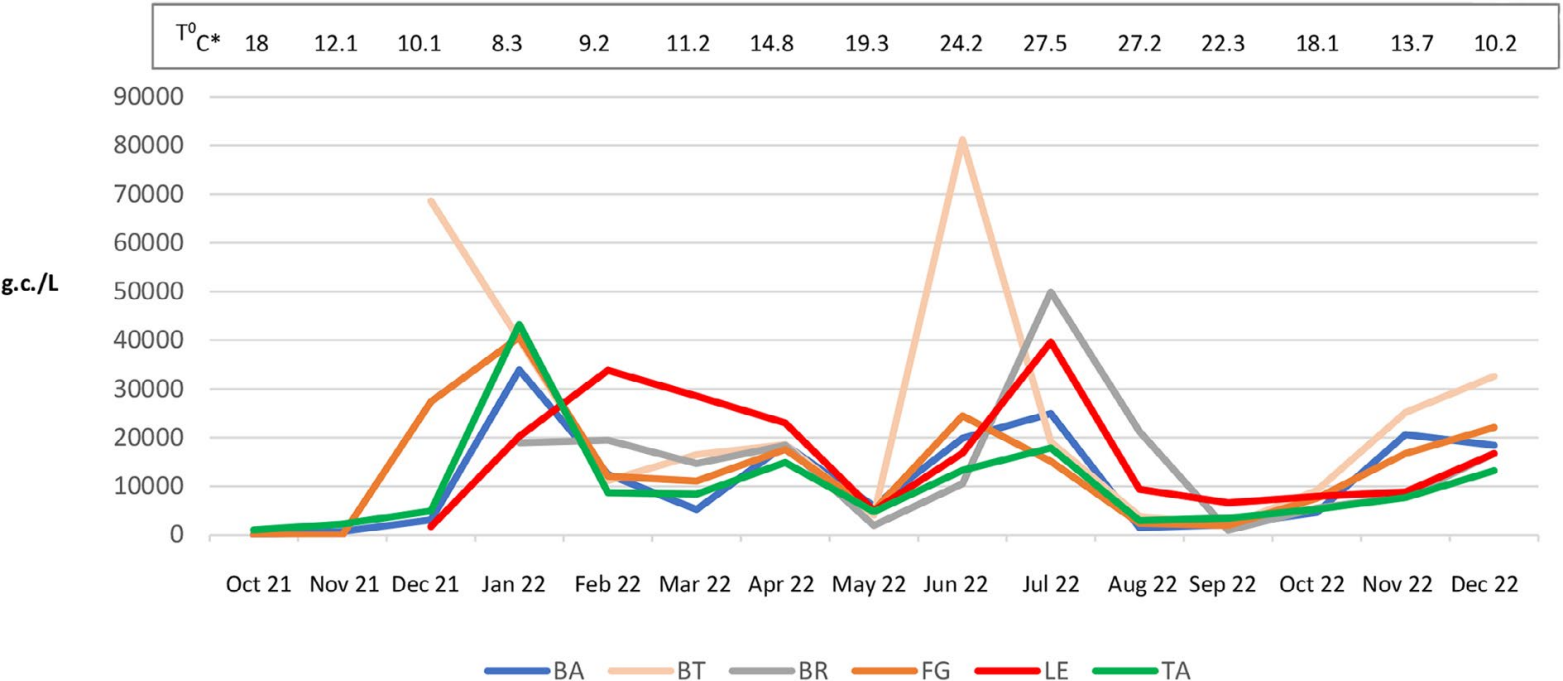
SARS-CoV-2 RNA detection (median value) in untreated wastewater samples using quantitative real-time reverse-transcription polymerase chain reaction for each month at wastewater treatment plants * Average monthly temperatures in the Apulia Region (Southern Italy) from October 2021 to December 2022 (Apulian Regional Civil Protection); BA, Bari; BT, Barletta-Andria-Trani; BR, Brindisi; FG, Foggia; LE, Lecce; TA, Taranto

In the majority of WWTPs for province, the highest viral loads were observed in the months of January, June, July, and December 2022 (Fig. 2). In the Supplementary Material, Figures S1–6 show the median values of SARS-CoV-2 RNA in untreated wastewater samples for each sampling month at the WWTPs distinguished by each province of the Apulia region (BA, BT, BR, FG, LE, TA).

**Table 1:** SARS-CoV-2 RNA detection in untreated wastewater samples, by WWTP and province

Province	WWTP	Median value g.c./L	Range g.c./L	n/N positive for SARS-CoV-2 (%)
BA	A	9000	627–254,000	49/53 (92.5)
	B	4330	39–106,000	98/103 (95.1)
	C	5250	30–133,000	94/99 (94.9)
	D	3910	157–72,500	51/53 (96.2)
	E	6210	43–212,000	50/52 (96.2)
BT	A	8050	527–60,100	51/52 (98.1)
	B	9000	112–98,600	49/51 (96.1)
	C	9405	314–148,000	46/52 (88.5)
	D	8600	437–975,000	48/52 (92,3)
BR	A	6600	106–97,800	49/51 (96.1)
FG	A	4450	188–78,400	51/51 (100)
	B	7200	555–77,600	50/54 (92,6)
	C	7550	218–72,000	46/47 (97.9)
LE	A	12,200	924–125,000	47/50 (94.0)
TA	A	5335	430–396,000	103/110 (93.6)
	B	5960	28–396,000	103/111 (92.8)
Total		6350	28–975,000	985/1041 (94.6)

BA, Bari; BT, Barletta-Andria-Trani; BR, Brindisi; FG, Foggia; LE, Lecce; TA, Taranto; n/N: number of positive samples/ total number of samples for each wastewater treatment plant (WWTP)

### SARS-CoV-2 Variant Trend Analysis

**Fig. 3:**
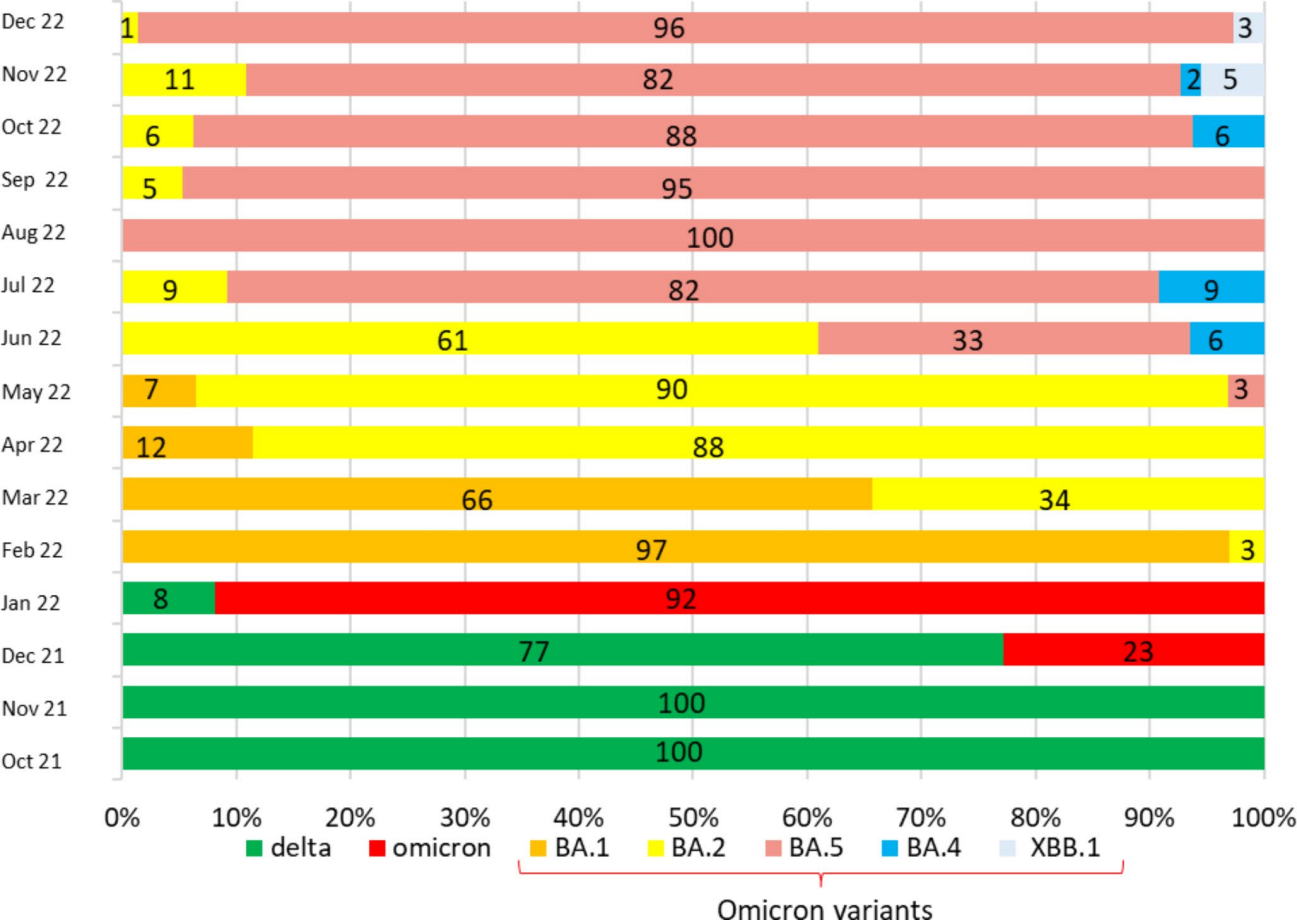
Distribution (%) of SARS-CoV-2 variants during October 2021 to December 2022 in the A pulia Region southern Italy

Figure 3 depicts the distribution of SARS-CoV-2 variants in positive sequenceable samples of wastewater from October 2021 to December 2022. In October and November 2021, our results confirmed the predominance of the Delta variant (B.1.617.2). In December 2021, the Omicron variant (B.1.1.159) was also detected and gradually spread; the variant was found in five WWTPs (BA-B, BA-C, BA-D, FG-A, FG-B) during 19–25 December 2021. By January 2022, the Omicron variant had become prevalent in comparison with the Delta variant, accounting for 92% of sequenceable samples whereas the Delta variant accounted for only 8%. After February 2022, the Omicron variant lineage BA.1 became predominant (97% of sequenceable samples); the Omicron variant lineage BA.2 accounted for the remaining 3%.

In March 2022, BA.1 was present in 66% of positive sequenceable wastewater samples, and BA.2 was present in 34%. In April 2022, BA.2 became predominant, present in 89% of positive sequenceable samples whereas BA.1 was present in only 11%. In May 2022, in addition to BA.1 (7%) and BA.2 (90%), the presence of BA.5 (3%) was detected. After June 2022, BA.1 disappeared, and the tested samples were positive for BA.2 (61%), BA.5 (33%), and BA.4 (6%). In July 2022, however, there was greater diffusion of BA.5 (82%), followed by BA.2 and BA.5 (9% each).

BA.5 was the only variant detected in August 2022, whereas in September 2022, wastewater samples were positive for both BA.5 (95%) and BA.2 (5%). As of October 2022, BA.5 was present in 88% of the samples tested, followed by BA.2 and BA.4 (6% each). In November and December 2022, in addition to the prevailing distribution of BA.5 (82% and 96%, respectively), BA.2 (11% and 1%, respectively), BA.4 (2% in November only), and XBB.1 (5% and 3%, respectively) were also detected.

### Temperature and Rainfall Data

The final Poisson regression model revealed the independent parameters (COVID-19 cases, median atmospheric temperature) that affected the dependent variable (SARS-CoV-2 RNA load in wastewater) (Table 2).

**Table 2:** Poisson regression model of SARS-CoV-2 load in untreated wastewater samples: final model

	β	(eβ − 1) = RR (%)	p-value
Intercept	0.07653		< 0.0001
COVID-19 cases in the 15 days after sampling	0.08188	8.53	0.04
Median atmospheric temperature on sampling day	−1.69	−81.54	0.03

RR, relative risk

The trend in COVID-19 cases during the 15 days following the day of sampling was directly proportional to the viral load. In the Supplementary Material, Figures S7–12 show the temporal distribution of SARS-CoV-2 RNA (median value) in untreated wastewater samples for sampling day and COVID-19 cases in the 15 days after sampling distinguished by provinces of the Apulia region (BA, BT, BR, FG, LE, TA). Conversely, an inverse correlation between SARS-CoV-2 load in wastewater samples and median atmospheric temperature was found on the day of sampling.

Regarding rainfall data, no influence on the SARS-CoV-2 load was found. This result was confirmed by comparing the median data of the viral load on rainy days versus non-rainy days. No statistically significant difference was found (7540 g.c./L vs. 7840 g.c./L; Wilcoxon rank-sum test with continuity correction, W = 24,436, p = 0.6983).

### Validation of Previous Predictive Model (De Giglio et al., 2021)

Validation of the predictive model (De Giglio et al., 2021) with updated datasets from the SARI project showed a sensitivity of 75.2% (421/560 positive samples for SARS-CoV-2), indicating at least 11 cases/100 000 inhabitants within 15 days of virus detection in wastewater, and a specificity of 100% (3/3 negative samples for SARS-CoV-2), indicating less than 11 cases/100,000 inhabitants within 15 days of sampling.

## Discussion

Unlike clinical surveillance, environmental surveillance offers an anonymous and non-invasive approach that allows for the rapid identification of COVID-19 clusters in a specific area (Daughton, 2020). In the Apulia Region, the COVID-19 pandemic has followed a trend that mirrors the national trend, with a succession of variations comparable to those detected in clinical patients. Specifically, in the months of January, June, and July 2022, a higher load of SARS-CoV-2 was detected in wastewater samples compared with other months.

It is known that different variants of the virus have varying levels of infectiousness and ability to evade the immune system. Compared with the Delta variant (B.1.617.2), Omicron variants BA.1 and BA.2 have greater capacity to evade the immune system (Cele et al., 2022; Liu et al., 2022). The BA.2 variant is approximately 1.5 times more infectious than BA.1 and 4.2 times more contagious than the Delta variant (Chen et al., 2022). The two most recently emerged variants, BA.4 and BA.5, share a high similarity with BA.2 and carry specific mutations in the spike protein. Studies have shown that after previous Omicron infection, BA.4 and BA.5 can escape antibodies present in the body, indicating evasion to previous immunity (Cao et al., 2022; Quandt et al., 2022). With spread of the highly transmissible BA.4/BA.5 sublineages, coupled with the gradual easing or lifting of social restrictions, the number of hospitalizations and deaths among vulnerable individuals has increased.

To date, few studies have investigated the potential impact of temperature on the SARS-CoV-2 viral loads in wastewater. Some authors (Markt et al., 2021) have demonstrated that storing wastewater samples at 4 °C for up to nine days has no significant effect on viral load whereas storing samples at − 20 °C leads to a significant reduction in the number of gene copies of SARS-CoV-2. It is possible that the presence and persistence of the virus in untreated wastewater, before it reaches a WWTP, may be influenced by environmental temperature conditions.

Our findings suggest an inverse correlation between the SARS-CoV-2 load in wastewater and median atmospheric temperature, indicating that the virus may persist longer in colder environments.

Hart and Halden (2020) used computational analysis and found that an ambient temperature of 20 °C leads to reduced detectability of SARS-CoV-2 RNA compared with lower ambient temperatures (5–10 °C). This is consistent with studies that report an optimal temperature of 8.72 °C for the peak of direct virus transmission (Mu et al., 2021). Consequently, cold environments would facilitate the survival and spread of the virus, contributing to the onset of an epidemic. In reality, to what extent higher ambient temperatures can prevent spread of the virus in the environment is not yet fully understood (Mu et al., 2021). A study conducted by Balboni et al. (2023) during the first and second waves of the pandemic in Italy demonstrated that a temperature of > 10 °C/50°F was associated with reduced rates of infections, hospitalizations, and deaths from COVID-19. Similarly, it has been suggested that the viral concentrations of SARS-CoV-2 in raw wastewater samples could be influenced by the sensitivity of the virus to high temperatures (Ahmed et al., 2020). This could explain why virus concentration in wastewater was generally higher in the winter season (January and December 2022) than in the summer season (August and September 2022). Several authors (Kumar et al., 2021; Paules et al., 2020) have reported that epidemics of emerging respiratory viruses, such as SARS (2002–2003), MERS (2012–2015) and the current COVID-19, originated in winter because they are strongly influenced by seasonality: sunlight, low winter temperatures, absolute humidity (dry season), host susceptibility to cold and seasonal changes in immunity. However, in our study, a significant increase in the load of SARS-CoV-2 in wastewater was observed in the summer season i.e. the months of June (24.2 °C), and July (27.5 °C) 2022. This deviation from results respect to the proposed correlation (low median atmospheric and high viral load) was probably due to the enormous influx of tourists in Apulia who may have been infected by the virus during the summer season. The removal of mandatory mask wearing and physical distancing, especially when associated with a low immune response and the spread of BA.4 and BA.5 sublineages, increased the risk of reinfection (Rahimi & Abadi, 2022). At the same time, however, the increase in viral load in January, could also be due to increasing of the gathering of individuals in closed spaces during the winter season, thereby increasing the risk of infection and transmission (Rahimi & Abadi, 2022). In our opinion, the role of temperature in the survival and/or replication of the virus remains unclear and requires further investigation.

The impact of rain on the SARS-CoV-2 load in wastewater also requires further investigation. Some studies have suggested that precipitation may reduce the sensitivity of WBE due to a diluent effect on the concentration of SARS-CoV-2 and consequently a substantial number of COVID-19 cases are needed to detect virus in wastewater (Lazuka et al., 2021; Saingam et al., 2023). Our findings showed that rain does not seem to affect viral loads but according to Lazuka et al., 2021, these data need to be contextualized considering also relationships between WWTP inlet flow rate and rainfall. Furthermore, the influence of rains should be analyzed considering the structure of the sewage system, the local and territorial characteristics, and the still poorly understood interaction of the viruses with the soil (Kumar at al., 2021; Lazuka et al., 2021).

Generally, it is important to acknowledge that other factors not accounted for in this study may influence the SARS-CoV-2 load in untreated wastewater, including the distance and time between the source and sampling site, the sampling time, the sewerage area, environmental parameters as the relative humidity, wind speed, and different transmissibility profiles of the various SARS-CoV-2 variants (Lazuka et al., 2021; Prasek et al., 2022; Prasek et al., 2023; Saingam et al., 2023; Solo-Gabriele et al., 2023).

Our predictive model proposed in the previous paper (De Giglio et al., 2021), which established at least 11 cases of COVID-19 per 100,000 inhabitants 15 days after the detection of SARS-CoV-2 RNA in wastewater was confirmed with this new data set for the period October 2021 - December 2022.

## Conclusions

The analysis of untreated wastewater provides valuable information regarding the prevalence of pathogens circulating in territorial and/or regional populations. Since the emergence of SARS-CoV-2, wastewater has been used as an epidemiological tool to carry out environmental surveillance, in support of clinical surveillance, to map the dynamics of the COVID-19 pandemic and observe the diffusion of new variants of SARS-CoV-2.

Our study in the Apulia Region supported the national data and confirmed the predictive model of COVID-19 cases previously developed. WBE can serve as an important and cost-effective tool, beyond the current pandemic, to detect the dynamics of other circulating viruses or newly emerged pathogens.

### Electronic Supplementary Material

Below is the link to the electronic supplementary material.

Supplementary Material 1

## Data Availability

Not applicable.
