# Supplementary material for: Wastewater-based Epidemiology and SARS-CoV-2: Variant Trends in the Apulia Region (Southern Italy) and Effect of Some Environmental Parameters
Source: Food Environ Virol. 2023 Sep 21;15(4):331–41. doi: 10.1007/s12560-023-09565-0 (PMC10654208; doi:10.1007/s12560-023-09565-0)
Supplement: Supplementary file 1 — Supplementary Material 1 [file 12560_2023_9565_MOESM1_ESM.docx]

SUPPLEMENTARY INFORMATION

**Wastewater-based epidemiology and** **SARS-CoV-2: variant trends in the Apulia Region (Southern Italy) and effect of some environmental parameters**

**Francesco Triggiano^1†^, Osvalda De Giglio*^1†^, Francesca Apollonio^1†^, Silvia Brigida^2^, Fabrizio Fasano^1^, Pamela Mancini^3^, Giusy Bonanno Ferraro^3^, Carolina Veneri^3^, Giuseppina La Rosa^3^, Elisabetta Suffredini^4^, Luca Lucentini^3^, Nicola Ungaro^5^, Giuseppe Di Vittorio^6^, Onofrio Mongelli^6^, Nelhudoff Albano^6^, Maria Teresa Montagna^1^**

^1^ Interdisciplinary Department of Medicine, University of Bari Aldo Moro, Piazza G. Cesare 11, 70124 Bari, Italy; [francesco.triggiano@uniba.it](mailto:francesco.triggiano@uniba.it) (F.T.); [osvalda.degiglio@uniba.it](mailto:osvalda.degiglio@uniba.it) (O.D.G.); [francesca.apollonio@uniba.it](mailto:francesca.apollonio@uniba.it) (F.A.); [fabrizio.fasano@uniba.it](mailto:fabrizio.fasano@uniba.it) (F.F.); [mariateresa.montagna@uniba.it](mailto:mariateresa.montagna@uniba.it) (M.T.M.).

^2^ Department of Biological and Environmental Sciences and Technologies, University of Salento, Campus Ecotekne, Monteroni di Lecce, 73047 Lecce, Italy; [silvia.brigida@unisalento.it](mailto:silvia.brigida@unisalento.it) (S.B.).

^3^ Department of Environment and Health, Istituto Superiore di Sanità, 00161 Rome, Italy; [pamela.mancini@iss.it](mailto:pamela.mancini@iss.it) (P.M.); [giusy.bonannoferraro@iss.it](mailto:giusy.bonannoferraro@iss.it) (G.B.F.); [carolina.veneri@iss.it](mailto:carolina.veneri@iss.it) (C.V.); [giuseppina.larosa@iss.it](mailto:giuseppina.larosa@iss.it) (G.L.R.); [luca.lucentini@iss.it](mailto:luca.lucentini@iss.it) (L.L.).

^4^ Department of Food Safety, Nutrition and Veterinary Public Health, Istituto Superiore di Sanità, 00161 Rome, Italy; [elisabetta.suffredini@iss.it](mailto:elisabetta.suffredini@iss.it) (E.S.).

^5^ Agency for the Environmental Prevention and Protection (ARPA Puglia), Corso Trieste 27, 70126, Bari, Italy; [n.ungaro@arpa.puglia.it](mailto:n.ungaro@arpa.puglia.it) (N.U.).

^6^ Department of Health Promotion and Animal Welfare, Apulia Region, Bari, Italy; [g.divittorio@regione.puglia.it](mailto:g.divittorio@regione.puglia.it) (G.D.V.); [n.albano@regione.puglia.it](mailto:n.albano@regione.puglia.it) (N.A.); [o.mongelli@regione.puglia.it](mailto:o.mongelli@regione.puglia.it) (O.M.).

^†^These authors contributed equally to this work.

***** Correspondence: [osvalda.degiglio@uniba.it](mailto:osvalda.degiglio@uniba.it); Tel.: +39-080-547-8476

**Figure S1**. Median value of SARS-CoV-2 RNA in untreated wastewater samples using quantitative real-time reverse-transcription polymerase chain reaction for each month at wastewater treatment plants – Province BA, Bari.

**Figure S2**. Median value of SARS-CoV-2 RNA in untreated wastewater samples using quantitative real-time reverse-transcription polymerase chain reaction for each month at wastewater treatment plants – Province BT, Barletta-Andria-Trani.

**Figure S3**. Median value of SARS-CoV-2 RNA in untreated wastewater samples using quantitative real-time reverse-transcription polymerase chain reaction for each month at wastewater treatment plants – Province BR, Brindisi.

**Figure S4**. Median value of SARS-CoV-2 RNA in untreated wastewater samples using quantitative real-time reverse-transcription polymerase chain reaction for each month at wastewater treatment plants – Province FG, Foggia.

**Figure S5**. Median value of SARS-CoV-2 RNA in untreated wastewater samples using quantitative real-time reverse-transcription polymerase chain reaction for each month at wastewater treatment plants – Province LE, Lecce.

**Figure S6.** Median values of SARS-CoV-2 RNA in untreated wastewater samples using quantitative real-time reverse-transcription polymerase chain reaction for each month at wastewater treatment plants – Province TA, Taranto.

**Figure S7**. Temporal distribution of SARS-CoV-2 RNA in untreated wastewater samples for each sampling day and COVID-19 cases in the 15 days after sampling - Province BA, Bari

**Figure S8**. Temporal distribution of SARS-CoV-2 RNA in untreated wastewater samples for each sampling day and COVID-19 cases in the 15 days after sampling - Province BT, Barletta-Andria-Trani

**Figure S9**. Temporal distribution of SARS-CoV-2 RNA in untreated wastewater samples for each sampling day and COVID-19 cases in the 15 days after sampling - Province BR, Brindisi

**Figure S10.** Temporal distribution of SARS-CoV-2 RNA in untreated wastewater samples for each sampling day and COVID-19 cases in the 15 days after sampling - Province FG, Foggia

**Figure S11**. Temporal distribution of SARS-CoV-2 RNA in untreated wastewater samples for each sampling day and COVID-19 cases in the 15 days after sampling - Province LE, Lecce

**Figure S12**. Temporal distribution of SARS-CoV-2 RNA in untreated wastewater samples for each sampling day and COVID-19 cases in the 15 days after sampling - Province TA, Taranto
